# Factors Related to the Continuity of Care and Self-Management of Patients with Type 2 Diabetes Mellitus: A Cross-Sectional Study in Taiwan

**DOI:** 10.3390/healthcare10102088

**Published:** 2022-10-20

**Authors:** Hsiao-Mei Chen, Bei-Yi Su

**Affiliations:** 1 Department of Nursing, Chung Shan Medical University, Taichung 40201, Taiwan; 2 Department of Nursing, Chung Shan Medical University Hospital, Taichung 40201, Taiwan; 3 Department of Psychology, Chung Shan Medical University, Taichung 40201, Taiwan; 4 Clinical Psychological Room, Chung Shan Medical University Hospital, Taichung 40201, Taiwan

**Keywords:** type 2 diabetes mellitus, continuity of care, self-management

## Abstract

Background: Most diabetic patients suffer from chronic diseases affecting their self-management status. This study aims to explore the relationship between the CoC and the self-management of patients with Type 2 Diabetes Mellitus (T2DM) and analyze the predictive factors affecting their self-management. Methods: Structured questionnaires were used for data collection. Convenient sampling was adopted to recruit inpatients diagnosed with T2DM in the endocrine ward of a medical hospital in central Taiwan. Results: A total of 160 patients were recruited. The average age of the patients is 66.60 ± 14.57 years old. Among the four dimensions of the self-management scale, the average score of the problem-solving dimension was the highest, and that of the self-monitoring of blood glucose was the lowest. The analysis results showed that the overall regression model could explain 20.7% of the total variance in self-management. Conclusions: Healthcare providers should attach importance to the CoC of T2DM patients and encourage patients to maintain good interaction with healthcare providers during their hospitalization. It is recommended to strengthen CoC for patients with diabetes who are single or with low educational levels in clinical practice to enhance their blood glucose control and improve diabetes self-management.

## 1. Introduction

Research on the care of people with diabetes not only pays attention to the changes in mortality or morbidity [1] but also gradually emphasizes that self-management closely relates to the maintenance of the physical and mental health of such patients [2,3]. Diabetes is a chronic epidemic disease in many countries. More than 37 million Americans suffer from diabetes, of which 90–95% have Type 2 Diabetes Mellitus (T2DM), mainly caused by being overweight, exercising less, and having an improper diet [4]. This high rate of prevalence is also found in Taiwan. According to the Taiwan Ministry of Health and Welfare (2022), diabetes ranks fifth among the top ten causes of death, with a standardized mortality rate of 23.8% per 100,000 among Taiwanese adults [5]. The annual prevalence of diagnosed type 2 diabetes is 8.30% [6]. Related studies have pointed out that various complications are caused by improper blood glucose control, including retinal-related complications (accounting for 72.6% of the patients), peripheral neuropathy (68.2%), and foot ulcers (12.7%) [7]. Related research has found that more chronic diseases of diabetic patients and longer time of the illness affect their self-management status [7,8], while their suffering from comorbidities is twice as higher as non-diabetic patients [9].

Continuity of Care (CoC) is a form of patient-centered care that patients and their families can obtain in a continuous and time-sensitive manner. In this form of care, medical resources are integrated through inter-professional teamwork, including physicians, nurses, nutritionists, physical therapists, occupational therapists, and pharmacists [10,11]. The CoC model provides complete care for diabetic patients [11] and improves patient care knowledge, the average length of stay, medical costs, and quality of life by evaluating and drawing up discharge plans, case management, or a CoC service pattern that combines both [10,12]. The Patient Continuity of Care Questionnaire (PCCQ) can be used to help professionals understand the nature of continuity of care to enable patients with chronic diseases to self-manage their conditions [12,13]. Koponen et al. indicated that if healthcare providers offer patient-centered autonomous diet, exercise, and medication support while patients have a sense of trust in the medical professional team, the trust of diabetic patients and the effectiveness of self-management will be improved [14].

Self-management refers to healthcare tasks and skills that individuals must undertake and perform daily under the guidance of a medical professional team to control or reduce the negative impact of diseases on physical health [15]. According to Shrivastava et al., diabetic patients have seven basic good self-management behaviors: healthy eating, active exercise, monitoring blood glucose, taking medication, good problem-solving skills, healthy coping skills, and risk reduction behaviors [16]. Lee et al. proposed four aspects of diabetes self-management: (1) communication with healthcare providers: patients can ask medical staff about relevant resources and adjust the treatment plan in time; (2) self-integration: patients can integrate diabetes care in daily life—for example, patients can control their diet in social situations; (3) self-monitoring of blood glucose: patients can set and record the blood glucose values to understand the situation of blood glucose control; (4) problem solving: patients can self-monitor their physical symptoms and make real-time decisions, such as confirming the conditions that cause blood glucose changes and dealing with them [2].

Although previous studies discussed the importance of self-management for diabetes, there is still limited research evidence on the relevance between CoC and self-management [15,17]. Therefore, the purpose of this study is (1) to assess the general information on sociodemographic characteristics, health status, CoC, and self-management of diabetic patients; (2) to explore the relationship between the sociodemographic characteristics, health status, and CoC of diabetic patients and self-management; (3) to analyze the important predictors of self-management for diabetic patients. Furthermore, the research results should offer a reference for clinical teams to provide patients with CoC to enhance their self-management knowledge and skills and promote health.

## 2. Materials and Methods

### 2.1. Research Design and Participants

The research proposal was reviewed and approved by the research institution’s Institutional Review Board (IRB) (No.109WFD2510093). It is a descriptive, correlational, and cross-sectional study. The data collection period was from 1 October 2020 to 16 April 2021 in the endocrinology ward of Chung Shan Medical University Hospital in Taiwan. Convenience sampling was used, and data collection was conducted before the patients were discharged from the hospital. The researchers had received standardized training. Subsequently, the data were collected after obtaining their consent. 

The inclusion criteria for the research participants included patients who (1) were 20–90 years old; (2) were inpatients diagnosed with T2DM; (3) could communicate in Mandarin Chinese and/or Taiwanese; (4) agreed to participate in the research and signed the consent form; (5) were willing to accept interviews and could fill out the questionnaire on their own or with the assistance of the researcher. On the other hand, the exclusion criteria excluded those patients with cognitive dysfunction or mental illness. 

A total of 165 questionnaires were distributed. For five invalid questionnaires which had too many missing values, they were omitted, and 160 valid questionnaires were collected. The recovery rate was 96.96%.

### 2.2. Measurement 

#### 2.2.1. Sociodemographic Characteristics

The sociodemographic characteristics of patients with T2DM include their age, gender, body mass index (BMI), marital status, living situation, religious belief, educational level, work status, and economic status.

#### 2.2.2. Health Status

The assessment of health status includes the number of diseases, complications, time of illness, treatment methods, A1C, frequency of foot examination, number of exercises per week, and smoking and drinking habits.

#### 2.2.3. Patient Continuity of Care Questionnaire

This study used the Chinese version of the Patient Continuity of Care Questionnaire (PCCQ) [12]. This questionnaire includes a total of 12 questions, including 2 subscales: Relationships with Providers in the Hospital and Information Transfer to Patients. Each question was scored on the Likert 5-point scale, ranging from 1 (Strongly disagree) to 5 (Strongly agree), and the total score of each question was averaged to obtain the score represented by each question. PCCQ has good reliability and validity, implying the scale’s good concurrent validity and internal consistency—the Cronbach’s α of all items was 0.960 [12]. Meanwhile, the Cronbach’s α value of this study’s PCCQ was 0.884, that of the relationships with providers in the hospital was 0.980, and that of the information transfer to patients was 0.962.

#### 2.2.4. Self-Management Scale

This study used the Chinese version of the Diabetes Patient Self-Management Scale, comprising four categories of communication with healthcare providers (6 items), self-integration (4 items), self-monitoring of blood glucose (5 items), and problem solving (5 items). There were 20 items to evaluate the overall self-management. Based on the Likert 4-point scoring, the average score of each category was 1–4 points, and the score range was 20–80 points. The higher the score, the higher the degree of self-management. The scale is remarkably good in terms of its convergent validity and internal consistency, having a Cronbach’s α value of 0.925 [2]. Further, the Cronbach’s α value of the overall self-management scale of this study was 0.910, while the category of communication with healthcare providers was 0.953, self-integration 0.945, self-monitoring of blood glucose 0.926, and problem solving 0.929.

### 2.3. Data Analysis

The SPSS 25.0 was used for statistical analysis. The mean of the variables was used to replace missing values. After the self-management regression model was selected, whether its residual items met the normal distribution was verified by the Kolmogorov–Smirnov (KS) test. *p* < 0.05 was set as the value for statistically significant correlation.

## 3. Results

### 3.1. Overview of T2DM, Sociodemographic Characteristics, Health Status, PCCQ, and Self-Management Scale

Most participants were female (51.2%) and over 65 (51.9%), with an average age of 66.60 ± 14.57 years. The majority of patients (41.9%) have a BMI score ranging from 18.5 to 23, 26.3 % from 24 to 26, and 25.6% higher than 27. Most of the patients’ educational level was below elementary school (41.3%), and 69.3% were unemployed. Regarding health status, most patients co-existed with more than three chronic diseases, with an average co-existence of 3.99 ± 1.78 diseases. Hypertension (75.0%), moderate to severe renal disease (45.6%), and hyperlipidemia (40.6%) were the most common diseases. In the 66.9% of patients had complications, the average complications were 1.39 ± 1.39. The majority of patients had diabetic complications (66.9%), followed by kidney complications (51.2%), eye (35%), and nerve (22.5%) lesions. The majority (48.8%) of the patients had suffered from the disease for more than 11 years (inclusive). Patients with well-controlled glycosylated hemoglobin (A1C 4–6.5%) accounted for 44.4%, while those with daily foot test > 1 time accounted for 22.5%. In total, 17.5% of patients had average weekly exercise > 3 times (each time > 30 min). Lastly, patients with smoking habits accounted for 14.4%, while those with drinking habits accounted for 10.6% (Table 1).

The average self-management score of the samples in this study was 50.34 ± 17.28. The problem-solving category scored the highest, while the average score for self-monitoring blood glucose was the lowest. In total, 45 (28.9%) patients never tested their blood sugar levels regularly, 38 (23.8%) patients had never told their healthcare providers that they wanted a diabetes control plan matching their daily routines, 42 (26.3%) patients had never recorded the blood glucose value to understand how far away they were from their goals, and 44 (27.5%) patients who had never set the target value for their A1C. 

The average PCCQ score was 51.94 ± 7.80, and the average for Relationship with Providers in Hospital and Information Transfer to Patients was 21.41 ± 3.48 and 30.53 ± 4.74, respectively (Table 2).

### 3.2. Correlation Analysis of Sociodemographic Characteristics, Health Status, CoC, and Self-Management of T2DM Patients

The older the patient, the worse the self-management of the communication with health providers (*r* = −0.283, *p* < 0.01), self-integration (*r* = −0.182, *p* < 0.05), and self-monitoring of blood glucose (*r* = 0.169, *p* < 0.05). Further, the higher the BMI value, the better the self-management of problem solving. Regarding marital status, self-management in terms of marriage and self-monitoring of blood glucose reached a significant correlation—the self-management of “married patients” was significantly better than that of “single patients”. The study also found that the self-management of communication between “patients without religious beliefs” and healthcare providers was significantly better than that of “patients with religious beliefs”. Regarding educational level, it had a significant correlation with self-management of communication with healthcare providers, self-monitoring of blood glucose, and problem solving (*p* < 0.01). The self-management of those with a “junior college degree or above” was significantly better than those with a “high (vocational) school or lower” education. There was a significant correlation in the patient’s cost of living with the self-management of self-integration (*F* = 3.191, *p* < 0.05). The self-management of those patients with “sufficient and surplus” cost of living was better than those with “somewhat inadequate/very inadequate” cost of living. Further, the main source of income was significantly correlated with communication with healthcare providers (*F* = 5.435, *p* < 0.01) of self-management. The self-management of patients with the main source of income from “work” was better than that from “children/spouse/siblings/parents” and “retirement/government subsidies”. In terms of communication with health providers (t = −2.874, *p* < 0.01), patients’ self-management was significantly correlated with whether they were working or not. Meanwhile, the communication with health providers of self-management of “working” patients was better than those who were “not working”.

Regarding health status, the number of chronic diseases significantly positively correlated with problem-solving self-management (*r* = 0.174, *p* < 0.05). The greater the total number of chronic diseases of the patients was, the better the self-management of problem solving. Patients’ complications were also significantly related to self-management. Those with “complications” were better than those with “no complications” in problem-solving self-management. The self-management in blood glucose monitoring (*F* = 3.422, *p* <0.05) of patients with A1C < 7% and well-controlled blood glucose status was better than those with A1C > 9%. The self-management of self-integration of patients with “exercise 1–2 times a week” was better than those with “weekly exercise <1 time”. This study found that patients’ CoC was positively correlated with their self-management, further showing that their overall self-management was better when the relationship between the patient and the healthcare providers during hospitalization or the transmission of information of CoC was better (Table 3).

### 3.3. Important Predictors Affecting Self-Management of Patients with T2DM

This study used multiple stepwise regression analyses to determine the primary factors affecting T2DM patients’ self-management. In addition, this study placed factors statistically significantly correlated with self-management into the regression model. The analysis results showed that marital status, educational level, and PCCQ were the predictive variables of self-management. PCCQ had the highest explanatory power, which could explain 13.5% of the variance, followed by marriage (2.3%) and educational level (4.9%). The above variables can effectively explain 20.7% of the total variance of the overall self-management of T2DM inpatients (*F* = 14.820, *p* < 0.001). The results indicated that after controlling the sociodemographic characteristics, health status, and CoC of T2DM inpatients, a significant correlation was found in self-management for T2DM patients who were “married”, had “college-level or above” education, or regarding their PCCQ (Table 4).

## 4. Discussion

### 4.1. Status of Self-Management and CoC of Patients with T2DM

This study found that 28.1% of patients had never regularly tested their blood sugar levels, and the average score of the self-monitoring of blood glucose was the lowest when the results of each category in the self-management scale were examined. Freeman pointed out that the failure of diabetic patients to monitor their blood glucose could easily lead to the risk of low blood glucose [17]. This problem also means that diabetic patients may lack relevant skills or seldom participate in self-care activities, resulting in poor self-management of blood glucose self-monitoring [2]. In addition, patients must have the ability to monitor blood glucose, performing routine blood glucose monitoring 2–3 times a week for blood glucose management [2,18,19]. Furthermore, it shows that healthcare providers must not only pay attention to diabetic patients’ self-monitoring of blood glucose but also their disease management knowledge and problem-solving ability [2].

In the self-integration category, the average score of self-integration ability, where patients would manage to control their blood glucose while participating in various social activities, was the lowest. Past studies have also suggested that diabetic patients choose or adjust their diet according to their living conditions and take appropriate actions according to the changes in blood glucose to improve their ability to integrate self-management [2,14].

In the problem-solving category, the average score of the number of blood glucose measurements was the lowest when patients were sick or stressed. Patients need to increase the number of blood glucose real-time measurements based on their body symptoms to effectively achieve the goals of disease control, prevention of disease deterioration, and self-management [20,21]. This finding is notably consistent with previous studies. Further, healthcare providers must guide patients to actively participate in the decision-making process of disease self-management according to their needs to reduce the risk of serious complications and effectively stabilize blood glucose levels. This can help patients improve their self-management ability [17,22,23].

In the context of the relationship, the scores were lower when the healthcare providers understood the patients’ expectations, beliefs, and preferences and when patients felt confident that the current healthcare providers would continue to take care of them after discharge. The studies of Koponen et al. [14] and Saint-Pierre et al. [11] have shown that the healthcare providers’ CoC of T2DM patients after their discharge from the hospital can ensure that the patients achieve and maintain ideal metabolic control and avoid complications. It has also been found by Hsieh et al. [13] and Koponen et al. [14] that the quality of diabetic patients’ CoC depends on the independent support of healthcare providers and their ability to maintain good communication with the patients, thereby improving the patients’ adherence of self-management.

### 4.2. Influencing Factors of Self-Management in Patients with T2DM

This study showed demographic characteristics and health statuses, such as age, BMI, marital status, religion, higher educational level, income, employment status, number of diseases, complications, HbA1C levels, weekly exercise habits, and PCCQ, were significantly correlated with self-management. This study found that the older the T2DM patients are, the poorer their self-management results will be. This finding is similar to the research results of Freeman [17] and Werfalli et al. [9]. The possible reason is that the treatment of older T2DM patients is more complicated, where older patients must face other disabilities related to aging. Hence, they are less involved in self-management education programs and lack knowledge about diabetes symptoms and complications, resulting in decreased self-management [9]. In this study, 26.3% and 25.6% of T2DM patients had BMI values of overweight and obesity, respectively, which is positively correlated with the problem-solving category score in self-management. Noteworthily, this result is consistent with the research results of Alodhayani et al. [24] and Whitehouse et al. [25]. Diabetic patients with heavier body weights are more aware of the relationship between blood glucose control and the changes in their physical symptoms. Thus, they have better problem-solving self-management capabilities, allowing them to test their blood glucose as soon as possible when they feel uncomfortable. In addition, this study found that the self-management of communication for “people without religious beliefs” with healthcare providers was significantly better than those “people with religious beliefs”. T2DM patients with religious beliefs may feel spiritually restrained, stressed, or guilty due to disease restrictions (e.g., diet) that affect their self-management ability to communicate with healthcare providers [26].

The self-management of T2DM patients was better among those with living expenses that were “abundant and surplus”, having main sources of income from “work”, or who were salaried workers. The results of this study are consistent with those of Boakye et al. [27] and Adhikari et al. [21]. When the patients’ annual income is higher, they have more opportunities to receive diabetes self-management education; hence, their self-management is better. Diriba et al. [28] also found that some patients with T2DM are engaged in full-time jobs to reduce the financial burden on their families. Thus, these patients have better self-management capabilities because their economic autonomy allows them to obtain more disease-related resources.

By controlling other factors, this study found that the self-management of “married persons” among T2DM patients was significantly better than that of “single persons”. This finding could explain the 2.3% variance of self-management in T2DM patients, matching the results of Alodhayani et al. [24] and Lundberg and Thrakul [26]. Most married T2DM patients live with their families. Hence, self-management is usually part of the family and social life, where family members and friends encourage and assist them.

The number of chronic diseases and complications is significantly positively correlated with self-management. In particular, patients with many chronic diseases and complications face more self-management challenges [3,17,20]. However, cooperating with healthcare providers and getting assistance in dietary choices and blood glucose monitoring can improve patients’ personal health and self-management ability [2,14].

This study also found that people with good blood glucose control (A1C < 7%) had better self-management in self-monitoring of blood glucose. As pointed out by related research, monitoring a patient’s blood glucose 2–3 times a week helps control A1C and recognize the deterioration of blood glucose control early, thereby improving the patient’s self-management [18,19]. Exercise is also an important factor affecting diabetes self-management [29]. This study found that patients who “exercised 1–2 times a week” had better scores in the category of self-integration in self-management. Developing a habit of doing physical activities helps patients lose weight and control blood glucose [9,29]. It is also recommended that diabetic patients participate in more than 2 h of exercise per week or perform resistance exercise 2–3 times a week, which will help improve the degree of self-management of diabetic patients [30,31].

Educational level was also a predictor of T2DM self-management. Self-management of T2DM patients with a junior college degree or above was better than illiterate/literate/primary school patients. Further, educational level is related to a patient’s acquisition of disease-related knowledge and self-care skills of execution and maintenance [3,24]. The A1C control effect and self-management level are also highly related [19]. Therefore, it is clinically recommended that a vivid, graphical, interactive, and positive feedback self-management education model be developed for those patients with lower educational levels to enhance their self-management ability [19,30].

The feature of this study is the inclusion of CoC variables, which helps examine the impact of CoC on the self-management of T2DM patients. Regression analysis shows that whether T2DM patients received CoC or not was the best predictor of the degree of self-management, which explained 13.5% of the variance. Strengthening the communication between T2DM patients and the healthcare providers and providing timely health information, emotional support, and response strategies that meet the needs of patients will enable them to form helpful habits. These habits include self-adjustment of diet, exercise, and blood glucose monitoring, integrating self-management into daily life [2,22], solving patients’ problems, and improving their level of self-management [2,3,22].

This study had some limitations. First, the sample size was limited, and it should be increased in the future to give us more statistical power to detect differences. Second, comprehensive inferences cannot be made restricted by the research design and the sources of research patients. However, the experimental design may be adopted if a more rigorous effectiveness evaluation can be carried out. Third, we collected data by utilizing subjective reports. Although it is a standard and direct measure of participants’ perceptions, differences between subjective reports and actual behavior might exist. Fourth, the time since diagnosis being six years or more reaches 70% of the research patients in this study. It is suggested that future research can investigate the situation of the continuity of care and self-management for the newly diagnosed T2DM. Nevertheless, this study can guide clinical practitioners in carrying out CoC for T2DM patients and as a reference for developing or designing self-management education modules.

## 5. Conclusions

Healthcare providers should emphasize the CoC of T2DM patients and encourage them to maintain good interaction with the healthcare providers during their hospitalization. Healthcare providers also need to provide individualized care, offer disease knowledge and emotional support, and improve patients’ trust in healthcare providers to strengthen the level of self-management of T2DM patients. It is also recommended that healthcare providers encourage single patients or those with an educational level below elementary school to participate in self-management education courses and provide multiple and innovative education models. In the policy aspect, a social welfare insurance system should be planned to provide relevant resources and strengthen patients’ economic capabilities to enhance their self-management.

## Figures and Tables

**Table 1:** Social characteristics and health status of patients (*N* = 160).

Variables	*N*	*%*
Sociodemographic characteristics		
Age		
Mean (SD)	66.60 (14.57)	
Gender		
Male	78	48.8
Female	82	51.2
BMI (kg/m^2^)		
Mean (SD)	24.61 (4.50)	
Marital status		
Single (unmarried/divorced/widowed)	51	31.9
Spouse (married/cohabiting, separated)	109	68.1
Living situation		
Solitary	8	5.0
Not alone	152	95.0
Number of people living in the household		
Mean (SD)	3.67 (1.96)	
Persons living with		
Spouse	85	52.1
Children	90	55.2
Grandchildren/parents/siblings/friends/ domestic helpers	60	36.8
Religion		
Without	109	68.1
With	51	31.9
Level of education		
Illiterate/literate (self-study)/primary	66	41.3
Junior high school/high school (vocational)	59	36.9
Junior college and above	35	21.9
Employment status		
Unemployed	113	69.3
Employed	50	30.7
Income		
Sufficient/more than sufficient	108	67.5
Roughly enough	18	11.3
Slightly insufficient/inadequate	34	21.3
Main source of income		
Children/spouse/siblings/parents	84	52.5
Pension/government grants	38	23.8
Employment	38	23.8
Health status		
Number of diseases		
Mean (SD)	3.99 (1.78)	
Complications		
Mean (SD)	1.39 (1.39)	
Time since diagnosis		
<5 year	48	30.0
6–10 years	34	21.2
11 years and above	78	48.8
Treatment methods		
No oral medicine	10	6.3
Oral medicine	78	48.8
Insulin therapy	45	28.1
Both Oral medicine and Insulin therapy	27	16.9
A1C levels		
A1C < 7%	71	44.4
A1C 7–9%	73	45.6
A1C > 9%	16	10.0
Frequency of foot examination		
<1 time per day	124	77.5
≥1 time per day	36	22.5
Number of exercises per week		
<1 time a week	121	75.6
1–2 times a week	11	6.9
>3 times a week	28	17.5
Smoking habit		
No	137	85.6
Yes	23	14.4
Alcohol use		
No	143	89.4
Yes	17	10.6

BMI: body mass index; A1C: glycosylated hemoglobin.

**Table 2:** Self-management, PCCQ scores in patients with diabetes.

Variables	Mean Score (SD)	Mean Item Score (SD)	Score Indicator
Self-management Total self-management score (20–80)	50.34 (17.28)	2.40 (0.81)	60.00
Communication with HCPs (6–24)	14.79 (5.76)	2.47 (0.96)	61.75
Self-integration (4–16)	10.36 (3.77)	2.59 (0.94)	64.75
Self-monitoring of blood glucose (5–20)	12.14 (5.19)	2.43 (1.04)	60.75
Problem solving (5–20)	13.04 (4.75)	2.61 (0.95)	65.25
PCCQ			
Total score of PCCQ (12–60)	51.94 (7.80)	4.33 (0.65)	86.60
Relationships with providers during hospitalization (5–25)	21.41 (3.48)	4.28 (0.70)	85.60
Information transfer to patients (7–35)	30.53 (4.74)	4.36 (0.68)	87.20

HCPs: healthcare providers, PCCQ: patient continuity of care questionnaire.

**Table 3:** Correlation between sociodemographic characteristics, health status, patient continuity of care, and self-management among patients with type 2 diabetes (*N* = 160).

Variables	Communication with HCPs	Self-Integration	Self-Monitoring of Blood Glucose	Problem Solving
Age ^a^	−0.283 **	−0.182 *	−0.169 *	−0.183
BMI ^a^	0.050	0.084	0.089	0.163 *
Marital status ^b^	−1.342	−1.513	−2.160 *	−1.249
Single (unmarried/ divorced/widowed)				
Spouse (married/cohabiting, separated)				
Religion ^b^	2.609 **	1.272	0.838	0.811
Without				
With				
Level of education ^c^	5.422 **	1.870	5.984 **	4.125 **
Illiterate/literate (self-study)/Primary				
Junior high school/high school (vocational)				
Junior college and above				
Income ^c^	2.504	3.191 *	1.806	3.605
Sufficient/more than sufficient				
Roughly enough				
Slightly insufficient/inadequate				
Main source of income ^c^	5.435 **	0.403	0.436	0.765
Children/spouse/brothers or sisters/parents				
Pension/government grants				
Employment				
Employment status ^b^	−2.874 **	−0.427	−0.413	−0.573
Unemployed				
Employed				
Health status				
Number of diseases ^a^	0.041	0.012	0.122	0.174 *
Complications ^b^	0.143	−0.009	−0.601	−2.255 *
No				
Yes				
HbA1C levels ^c^	2.203	1.254	3.422 *	2.022
HbA1C < 7%				
HbA1C 7–9%				
HbA1C > 9%				
Weekly exercise habit ^c^	1.651	5.292 *	0.815	0.330
<1 time a week				
1–2 times a week				
>3 times a week				
PCCQ ^a^				
Overall Relationships with providers during hospitalization	0.389 ** 0.341 **	0.236 ** 0.281 **	0.335 ** 0.321 **	0.338 ** 0.307 **
Information transfer to Patients	0.388 **	0.181 *	0.315 **	0.330 **

* *p* < 0.05, ** *p* < 0.01. ^a^: Pearson correlation, ^b^: *t*-test, ^c^: F-test, HCPs: healthcare providers, BMI: body mass index, PCCQ: patient continuity of care questionnaire.

**Table 4:** Important predictive variables of the self-management of patients with type 2 diabetes (*N* = 160).

Variables	Self-Management					
Predictor	B	SE	Beta	Adjust R^2^	*t*	*p*
Marital status						
Spouse (married/cohabiting, separated)	6.111	2.615	0.165	0.023	3.352	0.001 ***
Single (unmarried/divorced/widowed) (reference group)						
Level of education						
Junior college and above	9.882	2.948	0.237	0.049	3.352	0.001 ***
Illiterate/literate (self-study)/Primary (reference group)						
PCCQ	0.827	0.157	0.370	0.135	5.227	0.001 ***

Linear regression was used for data analysis. B: unstandardized regression coefficient, PCCQ: patient continuity of care questionnaire, adjust R²: 0.207, F: 14.820, *** *p* < 0.001.

## Data Availability

The data supporting this study’s findings are available on request from the corresponding author.
